# Healthy Lifestyle Behaviors and Their Association with Self-Regulation in Chilean Children

**DOI:** 10.3390/ijerph17165676

**Published:** 2020-08-06

**Authors:** José Francisco López-Gil, Xavier Oriol-Granado, Mikel Izquierdo, Robinson Ramírez-Vélez, Omar Fernández-Vergara, Jordi Olloquequi, Antonio García-Hermoso

**Affiliations:** 1 Departamento de Actividad Física y Deporte, Facultad de Ciencias del Deporte, Universidad de Murcia (UM), 30720 San Javier, Region of Murcia, Spain; josefranciscolopezgil@gmail.es; 2 Facultad de Educación y Ciencias Sociales, Universidad Andres Bello, Fernández Concha 700, Las Condes, Santiago 7550196, Chile; xavier.oriol@unab.cl; 3 Navarrabiomed, Complejo Hospitalario de Navarra (CHN), Universidad Pública de Navarra (UPNA), IdiSNA, 31008 Pamplona, Navarra, Spain; mikel.izquierdo@gmail.com (M.I.); robin640@hotmail.com (R.R.-V.); 4 GICAEDS Group, Faculty of Physical Culture, Sport and Recreation, Universidad Santo Tomás, Bogotá 110311, Colombia; 5 Laboratorio de Ciencias de la Actividad Física, el Deporte y la Salud, Universidad de Santiago de Chile, USACH, Santiago 71783-5, Chile; omar.fernandez@usach.cl; 6 Instituto de Ciencias Biomédicas, Facultad de Ciencias de la Salud, Universidad Autónoma de Chile, Talca 3460000, Chile; jolloquequig@uautonoma.cl

**Keywords:** social–emotional development, physical activity, screen time, Mediterranean Diet

## Abstract

Background: Self-regulation comprises a series of important competencies, such as the ability to control inner states or responses toward thoughts, attention, emotions, or even performance. The relationship between self-regulation and different healthy lifestyle behaviors among children has not been examined in depth to date. The aim of this study was to explore the association between physical activity, screen time levels, and/or Mediterranean Diet adherence and self-regulation in Chilean children. Methods: A total of 1561 children aged 8–12 years from eight public schools with low socioeconomic status were included. Physical activity, screen time, Mediterranean Diet, and self-regulation were assessed with validated questionnaires. Results: Children who were classified as active or those who reported less than 2 h per day of screen time had higher self-regulation than those who were classified as inactive or counterparts with 2 h per day or more of screen time, respectively. Using joint categories, active children both with low and high screen time showed higher self-regulation compared to inactive/high screen time peers. Additionally, active groups with adherence or non-adherence to the Mediterranean Diet had higher self-regulation compared to inactive and non-adherence peers. Conclusion: Having a greater number of healthy habits, mainly regular physical activity, was associated with higher self-regulation, which might be one potential strategy to promote child social-emotional development.

## 1. Introduction

Self-regulation (SR) is defined as psychological conduct which comprises a series of important competencies, such as the ability to control inner states or responses toward thoughts, attention, emotions, or even performance [1]. A set of developing regulatory processes seems to be essential to individual differences in personality development and behavioral modification [2], and this includes biological factors, including neurobiology; physiology, or genes; and situational elements, such as parenting, experience, and environment [3].

Following this line, preadolescence is a period in which children increase their autonomy in health behaviors (e.g., eating, exercise habits), and many of them require SR [4]. Thus, childhood and adolescent SR is becoming increasingly important as a target of intervention, due to the growing evidence of its positive associations with health, social, and educational outcomes [5]; and due to the changing ability to exercise cognitive control of beliefs, emotions, and behaviors, it is one of the most serious alterations from infancy to early childhood [6].

Poor SR, for example, the inability to harness cognitive, emotional, and motivational resources to achieve goals, is hypothesized to contribute to unhealthy behaviors throughout life [4]. Thus, regular participation in physical activity (PA) is crucial for physical and cognitive function at any age because insufficient activity may result in several physical and psychological illnesses. On the other hand, it has been suggested that screen time might replace the time that could be spent on other activities and tasks which foster cognitive, behavioral, and motor development [7]. Prior literature has suggested that excessive screen time (ST) exposure is linked to later child self-regulatory difficulties [8].

On the other hand, adherence to the Mediterranean Diet (MD) is another healthy lifestyle habit, recognized worldwide for helping to prevent and control chronic non-communicable diseases over the entire lifespan [9]. In this sense, an important review has highlighted the importance of fostering the MD in childhood and adolescence in order to promote positive physical, cognitive, and academic development [10]. Although there is a belief that the MD is exclusive to Mediterranean countries, there are four other Mediterranean-type ecosystems, located between the parallels 30° and 45° of northern or southern latitude with their coasts facing west, including the central region of Chile [10]. Likewise, agricultural production in the central area of our country includes products similar to those of Mediterranean countries [11]. Thus, it has been pointed out that Chile has all the necessary elements to implement this type of diet [12].

Considering the above-mentioned evidence, exploring the relationship between SR and different healthy lifestyle behaviors could be useful for further purposes: First, for a better understanding of the mechanisms underlying engagement in multiple health behaviors; second, for creating and establishing interventions integrating multiple components of SR development, as it seems to be more successful in decreasing health risk behaviors and encouraging positive health behaviors; and third, because most of the studies on SR have been performed in high-income countries [13]. Thus, exploring these associations in Chile, a low-to-middle income country, could have a special relevance.

We hypothesized that children who have healthy lifestyle behaviors (e.g., achieving higher levels of PA, lower ST, and higher adherence to the MD) would have higher SR scores compared to those who present unhealthier behaviors. Thus, the aim of the study was to explore the association between PA, ST levels, and/or MD adherence and SR in Chilean children.

## 2. Material and Methods

### 2.1. Participants

Details of this study were published elsewhere [14]. In the present study, a total of 1561 children aged 8–12 years old were included from eight public schools with low socioeconomic status in the district of Santiago (Chile). Approval to conduct the study was granted by the ethics committee of the University of Santiago (Code number: 938). All children’s parents signed consent forms and received a full verbal and written explanation of the aim of the study and its anonymous nature.

With regard to the criteria for exclusion, some children were excluded on the basis of the following: (a) children who suffered from some kind of dysfunction that limited the practice of physical activity (i.e., any disease or motor problem); (b) children who had not resided in Santiago (Chile) for at least one school year; (c) children who were taking some kind of pharmacological treatment. The exclusion of participants of the study was carried out a posteriori, without the children being aware of their exclusion, in order to avoid undesirable situations.

### 2.2. Data Collection Method

A group of trained investigators conducted all measures under standardized conditions, with the pupils in a quiet classroom in the presence of a teacher at all times. All of the questionnaires were filled by the participants with the help of investigators.

#### 2.2.1. Self-Regulation (SR)

SR was determined using the adapted Moilanen’s inventory [15], which was developed by Children’s Worlds: International Survey of Children’s Well-Being [16]. This scale is composed of five items related to short-term regulation (i.e., monitoring, persevering adapting, activating, and inhibiting), specifically to emotional regulation and immediate objectives, for example, “When I’m sad, I can usually start doing something that will make me feel better,” or “After I’m interrupted or distracted, I can easily continue working where I left off.” Responses were provided on a 5-point Likert scale with options ranging from 1 (never) to 5 (very often). The total score of the scale was calculated by the average score of all items, with possible scores ranging from 0 to 5. Higher scores represent greater SR. Internal consistency was 0.82.

#### 2.2.2. Physical Activity (PA)

The measurement of PA was based on the question formulated by Prochaska et al. [17] as follows: “Normally: how many days were you physically active for a total of at least 60 min in the last week?” (response options ranged from 0 to 7 days per week). Those who met 60 min of PA all days of the week were considered active children. Internal consistency was 0.77 [17].

#### 2.2.3. Screen Time (ST)

ST was determined by asking children to report the number of hours of screen time per typical day in the past 7 days. The following question was asked: “On an average school day, how many hours do you watch TV, play video or computer games or use a computer for something that is not school work?” Internal consistency was 0.72. ST was categorized into optimal use (less than 2 h/d) and inadequate use (at least 2 h/d), according to the American Academy of Pediatrics [18].

#### 2.2.4. Mediterranean Diet (MD) Adherence

The Mediterranean Diet Quality Index for Children and Adolescents (Kidmed) questionnaire [19] was used to determine dietary patterns. The Kidmed score ranges from −4 to 12 points, and adherence to the MD was considered when children scored ≥8 points.

#### 2.2.5. Covariates

Sleep quality—The Spanish version of the Sleep Self-Report questionnaire was used to assess sleep habits [20].

Anthropometric measures. All measures were carried out at the same time in the morning, between 8:00 and 12:00. A portable electronic scale was used to measure body mass (Seca 769, Hamburg, Germany), with students barefoot and wearing light clothing. Height was measured using a portable stadiometer (Seca 220, Hamburg, Germany). BMI (body mass/height^2^) z-score was calculated using the International Obesity Task Force age-specific and sex-specific cutoffs [21].

### 2.3. Statistical Analysis

Descriptive data are presented as means and standard deviation for continuous variables and frequencies and percentages for categorical variables. The SR variable satisfied the tests of homoscedasticity (Levene variance homogeneity test) and normality (Kolmogorov–Smirnov test) of their distributions (*p* > 0.05).

Preliminary analyses showed no significant interactions between sex and mean differences in PA, ST, and MD (PA *p* = 0.611; ST *p* = 0.666; and MD *p* = 0.373); therefore, all analyses were performed with boys and girls together to increase statistical power. Partial correlations were used to determine the relationship between variables. An analysis of covariance (ANCOVA) was used to estimate differences between mean values of SR across PA (active or inactive), ST (low or high), and MD (adherence or non-adherence) categories. Additionally, ANCOVA with pairwise post-hoc comparisons using Bonferroni was used to test the differences between mean values of SR across combined healthy lifestyle categories, using four groups for each combination: (1) PA and ST (inactive and ≥2 h/d, inactive and <2 h/d, active and ≥2 h/d, and active and <2 h/d); (2) PA and MD (inactive and non-adherence, inactive and adherence, active and non-adherence, and active and adherence); (3) ST and MD (≥2 h/d and non-adherence, ≥2 h/d and adherence, <2 h/d and non-adherence, and <2 h/d and adherence). Finally, differences in mean values of SR according to the number of healthy habits that children met were also explored. All analyses were adjusted by age, sex, BMI z-score, and sleep quality. Data analyses were performed using the Statistical Package for Social Sciences (Version 23.0) software. *p* < 0.05 was considered statistically significant.

## 3. Results

Characteristics of the children are listed in Table 1. The mean (SD) age of the children was 10.16 (1.21) years old, and the sample was primarily boys (65.4%).

Table 2 shows partial correlations between SR and PA, ST, and MD. PA (*r* = 0.265; *p* < 0.001) and MD (*r* = 0.091; *p* = 0.030) positively correlated with SR, and ST was negatively related to this outcome (*r* = −0.125; *p* = 0.003).

**Figure 1:**
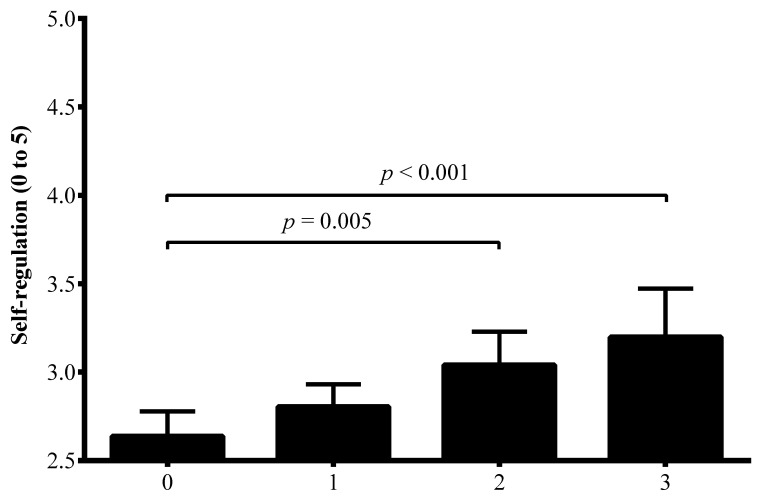
Differences in mean values of self-regulation according to the number of healthy lifestyle habits that children met.

Figure 1 depicts that children who met two (*p* = 0.005) or three (*p* < 0.001) healthy lifestyle behaviors had higher SR than children who did not meet any of them.

**Figure 2:**
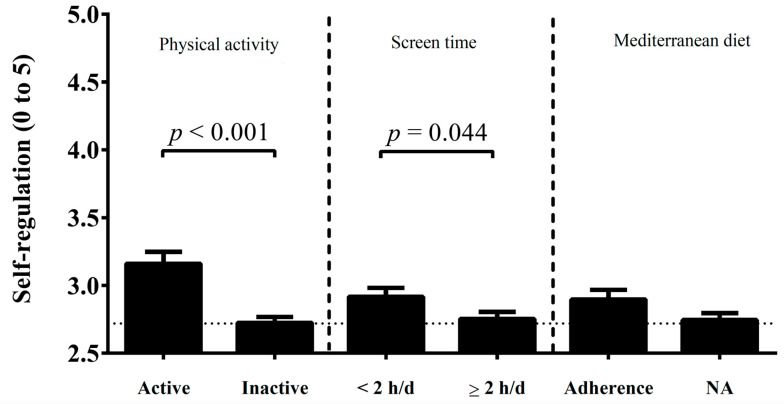
Differences in self-regulation according to physical activity, screen time, and Mediterranean Diet categories. NA, non-adherence to the Mediterranean Diet.

Figure 2 shows the differences in SR according to PA, screen time and MD categories. Children who are classified as active had higher SR than inactive counterparts (*p* < 0.001). Also, children who reported less than 2 hours per day of ST had higher SR than counterparts with 2 hours per day or more per day (*p* = 0.044). 

**Figure 3:**
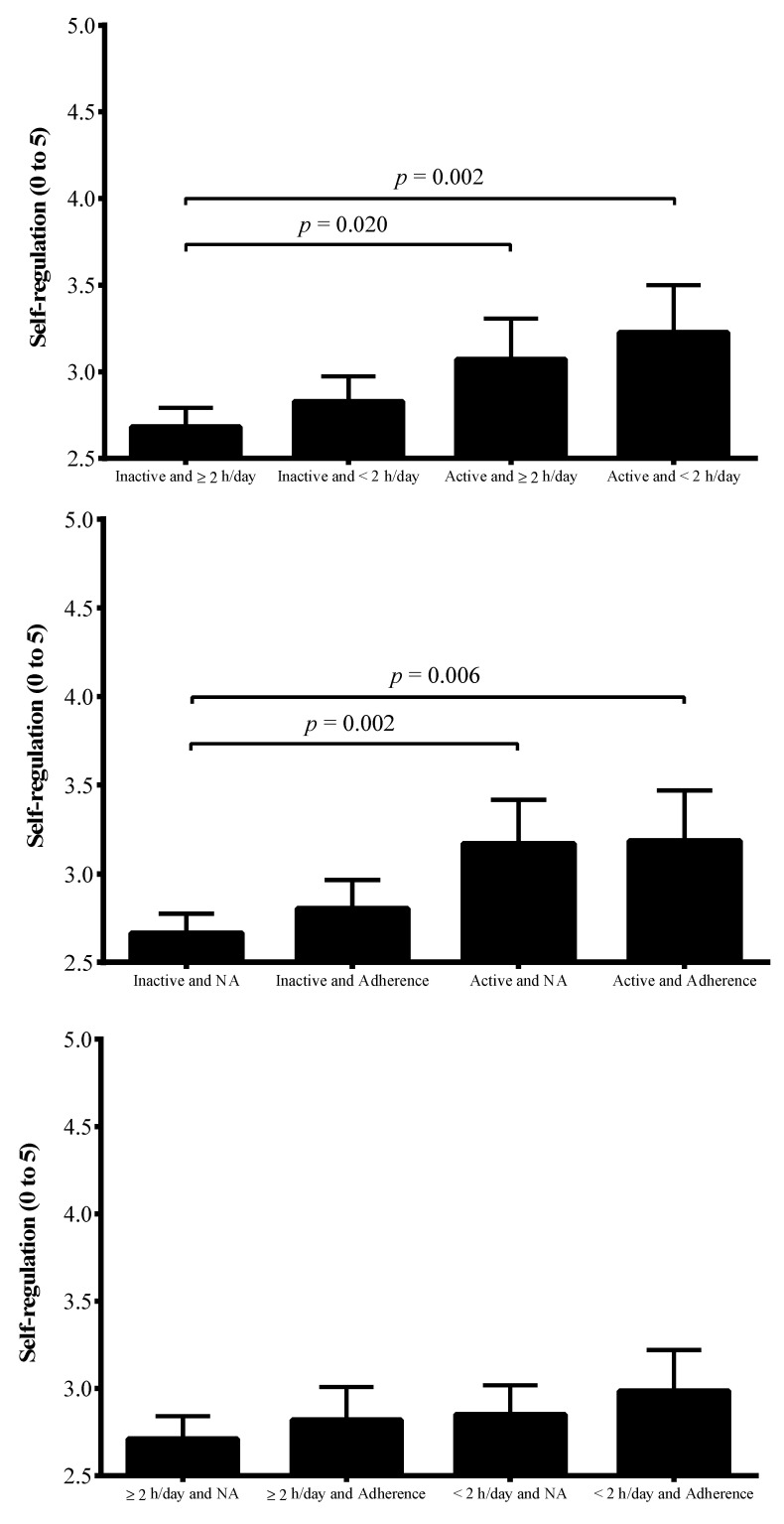
Differences in mean values of self-regulation across combined groups of physical activity, screen time, and Mediterranean Diet. NA, non-adherence to the Mediterranean Diet.

Finally, Figure 3 shows the differences in mean values of SR across combined groups of PA, ST, and MD. Active groups both with low (*p* = 0.002) and high ST (*p* = 0.020) showed higher SR compared to the inactive/high ST group. Further, active groups with adherence (*p* = 0.006) or non-adherence (*p* = 0.002) to the MD had higher SR compared to inactive and non-adherence peers.

## 4. Discussion/Conclusions

Although some studies have examined the association between SR and different healthy lifestyle habits, only a few of them have evaluated these associations in schoolchildren. Overall, meeting a higher number of healthy lifestyle habits was associated with higher SR. Specifically, current analyses show that children considered active and who reported less than 2 h of ST per day had higher levels of SR, this being the case when these positive behaviors were present in a joint manner.

Recent reviews have suggested that promoting PA might protect mental health in youths [22,23]; however, most of the studies included in these reviews are focused on adolescents. Our study, focused on children aged 8–12 years old, suggested that children who are classified as active had higher SR than inactive counterparts. In line with our findings, participating in PA has been pointed out as an opportunity to increase SR and coping skills that may exert positive effects on mental health [24]. Likewise, in a recent meta-analysis performed by Pandey et al. [5], it was reported that most of the interventions using PA or exercise to stimulate SR achieved higher scores compared to intervention groups. Despite the fact that the mechanisms responsible for the effects of PA on SR are unclear, it could be hypothesized that increased PA levels help to build personal resources such as social connectedness and autonomy [25], self-acceptance [24], positive affect, and life satisfaction [14]. People with these resources are more capable of effectively coping with daily-life challenges and maintaining control over environmental circumstances [1].

Conversely, we found that excessive time spent in ST is inversely associated with SR. Although it is not clear how the changes in sedentary behavior may influence both executive functions and SR processes, which are vital for the long-term maintenance of a healthy behavior regimen [26], it has been shown that an increase in ST could displace the time spent engaging in PA (displacement theory) [27], which could indirectly lower SR levels. It also seems possible that lower levels of ST may contribute to fewer later social–emotional difficulties (e.g., externalizing behaviors, executive functioning deficits, and aggression) [8]. In this respect, while reducing sedentary time is likely to have meaningful implications for a greater number of psychosocial outcomes [22], it is highly probable that the activity with which sedentary behavior is replaced (i.e., sleep, light activity, moderate-to-vigorous PA) influences the type and degree of these effects [26]. Therefore, decreasing the quantity of screen media exposure could be one strategy to address SR difficulties in children [28].

On the other hand, although we did not find statistically significant differences between MD adherence and SR score, some studies suggested that following a healthier diet was also associated with several benefits among young populations [10]. However, when high MD adherence was shown in combination with meeting the PA guidelines and without excessive ST hours, the SR was higher. This result agrees with the study performed by Fleig et al. [29], who suggested that those who have high levels of transfer cognition are more likely to engage in healthy dietary behaviors. Likewise, in another study, the clustering of PA and fruit and vegetable intake was suggested as an indicator of potential transfer of SR strategies [30]. These results could be justified by theory-based mechanisms of cross-behavior regulation, which refers to the regulation of at least two health behaviors, such as following a healthy diet while practicing PA on a regular basis [31].

Our findings also reveal that SR seems to exert a crucial role in achieving healthy lifestyle habits, i.e., it is also possible that this association could be bidirectional (e.g., child SR behaviors may predict child ST exposure). This fact could be explained by the interesting implications of habit development in the context of multiple healthy lifestyle behaviors: (i) As habit strength increases, the set of available SR resources rises as behavior promotion becomes more effective; and (ii) if people follow a PA routine rather effortlessly, SR resources occupied by specific exercise demands become unoccupied and can be invested in other behavioral tasks, such as diet planning [32]. On the other hand, it has been suggested that some intervention programs focused on diet and PA in children and adolescents have limited effectiveness [33]. One of the reasons for this may be the limited attention paid to the basic mechanisms of behavioral change in health [34], specifically the SR processes that may determine if the promoted health behaviors are implemented. In this sense, SR can be developed both in childhood and adolescence, providing an extended window of opportunity to intervene and improve outcomes for politicians, health professionals, and educators focusing on prevention, since SR attracts more attention as an intervention target [5].

Some limitations and strengths should be recognized. First, the cross-sectional design of the data limits the causal inference between healthy lifestyle habits and SR, i.e., there may be an inverse causality if children with low SR are less prone to follow healthy lifestyle habits. However, the proper analysis of cross-sectional data represents a useful first step in detecting relationships between these behaviors and SR in Latino schoolchildren. Nevertheless, these results need to be confirmed in longitudinal and interventional researches. Second, the participants were selected only within one area of the city of Santiago (Chile). Third, self-report questionnaires were used to assess PA, ST, and diet in this study. Finally, the sample of our study had no parity between sexes, since there was a higher percentage of boys than girls. The most remarkable strength of the present study was the use of a standardized and validated measuring instrument for evaluating SR in a large sample of children from 16 countries, according to Children’s Worlds: International Survey of Children’s Well-Being [16].

In conclusion, our study demonstrated that children who meet the PA guidelines and have low ST are separately and jointly associated with SR. Similarly, having a greater number of healthy lifestyle habits was associated with higher SR, which might be one potential strategy to promote child social–emotional development. In this sense, our results seem to suggest that PA, in conjunction with other lifestyle habits, could improve personal resources in order to maintain the energy needed for self-regulatory processes. It is important to note that for children, it is especially complicated to regulate impulses and maintain socially correct behavior [1]. For this reason, healthy lifestyles, such as regular PA, limited ST, and healthy nutrition promotion, should not be pursued in isolation, but rather as a part of a larger promotion of the importance of engaging in healthy lifestyle behaviors during childhood [35].

## Figures and Tables

**Table 1:** Participants’ characteristics (*n* = 1,561).

Age, years	10.16 (1.21)
Girls, n (%)	540 (34.6)
Weight, kg	42.58 (11.10)
Height, m	1.42 (0.09)
BMI, kg/m^2^	20.99 (4.06)
BMI z-score	1.26 (1.17)
Overweight/obese, n (%)	651 (41.7)
Physical activity, days/week	3.82 (2.27)
Screen time, h/day	3.05 (1.67)
Mediterranean Diet (−4 to 12)	6.32 (2.20)
Sleep quality (0 to 32) *	10.57 (5.66)
University level, n (%)	
Mother	264 (16.9)
Father	306 (19.6)
Self-regulation (0 to 5)	2.72 (0.89)

Data are mean (SD) or number and proportions (%). * Sleep Self-Report questionnaire [20].

**Table 2:** Partial correlations between self-regulation with physical activity, screen time, and Mediterranean Diet.

	Self-Regulation	Physical Activity	Screen Time
Physical activity	0.265 **		
Screen time	–0.125 *	−0.075 *	
Mediterranean Diet	0.091 *	0.131 *	–0.052

Data are mean (SD). Analyses were fully adjusted by age, sex, body mass index z-score, and sleep quality. * *p* < 0.01; ** *p* < 0.001.
